# Developing and evaluating a Portuguese-language meditation App for medical students: motivation, adherence, and emotional effects

**DOI:** 10.3389/fpsyg.2025.1422205

**Published:** 2025-03-05

**Authors:** Ana Rita Soares, Sandra Soares, Tânia Brandão, Ricardo João Teixeira, Isaura Tavares

**Affiliations:** ^1^Department of Biomedicine, Unit of Experimental Biology, Faculty of Medicine, University of Porto, Porto, Portugal; ^2^Dr. Manuel Gomes de Almeida School Cluster – AEMGA, Vila Nova de Gaia, Portugal; ^3^William James Center for Research, ISPA – Instituto Universitário, Lisbon, Portugal; ^4^University of Coimbra, Faculty of Psychology and Educational Sciences, CINEICC, Coimbra, Portugal; ^5^REACH - Mental Health Clinic, Porto, Portugal; ^6^I3S-Instituto de Investigação e Inovação em Saúde, Universidade do Porto, Porto, Portugal

**Keywords:** stress, anxiety, mindfulness, meditation, self-care, well-being, health, online resources

## Abstract

The well-being of medical students is affected by high stress levels. The relevance of a mindfulness mediation app (Med@Med) specifically produced to help medical students at a Portuguese medical school cope with stress was evaluated. The app, consisting of 21 short meditations, was totally developed in Portuguese to fulfill with the needs of some of the students. The motivations to use the app, adherence to it, and its emotional benefits were evaluated. A total of 147 medical students were enrolled in the study. Students completed questionnaires related to emotional regulation (Emotion Regulation of Others and Self) and emotional thermometers before the first meditation and at the end of the project. Before and after each meditation, the students were invited to identify their basic emotion (joy, fear, disgust, anger, or sadness), no perceived emotion, or no reply. Participants received daily motivational messages (scientific or in lay language) or no message during the first 7 days of the project and the retention was registered. The main motivations to use the Med@Med app were to experience meditation (33%), decrease stress/anxiety (25%), or implement a daily meditation routine (16%). The remaining motivations of the students were sleep improvement or enhancement of academic performance. The self-motivation to use the app was high (7.3 ± 3.2 on a 1–10 scale). Participants that received daily messages in lay language, which summarized scientific findings about the benefits of meditation, were more likely to continue to use the app. The emotions changed from pre- to post-practice, with an increase in self-identification with joy and decrease of fear and sadness. An improvement in the intrinsic emotional regulation subscale (*p* < 0.01) was detected. In comparison with age-matched students that did not use the Med@Med app, the students that used the app presented less emotional distress and anxiety as evaluated by emotional thermometers. This study shows that medical students are motivated to use a meditation app. The improvement of emotion-related parameters after the use of the Med@Med app is a promising result. The benefits of using the Med@Med meditation app prepared in Portuguese keeping in mind the needs of medical students should be evaluated in other Portuguese-speaking medical schools.

## 1 Introduction

Several systematic reviews and meta-analyses have pointed out that medical students throughout the world are at risk of developing mental health issues (Dyrbye et al., 2006; Rotenstein et al., 2016). High stress levels were reported among medical students, both in self-perceived stress and in more objective measures, such as cortisol levels (Hearn and Stocker, 2022). The demands of the medical course likely contributes to the stress of medical students higher than in other courses, even in the first year of the medical course (Jordan et al., 2020). Sustained stress may lead to mental health issues, such as anxiety and depression, leading to suffering in students and impacting their future medical practice. An association between high stress/anxiety and lower academic performance and decreased efficacy of clinical practice was also reported (Bramness et al., 1991). Severe sadness, suicide, or suicide-related thoughts among medical undergraduate students need to be taken note of Rotenstein et al. (2016).

Stress management and mental health issues have been widely discussed in the context of medical education (Carrieri et al., 2020). A recent systematic review of review-level evidence of interventions in university students, including medical students, showed that mindfulness-based interventions (MBI) reduce common mental health challenges (Worsley et al., 2022). Mindfulness meditation is aligned with the Western practices to manage stress, namely by the implementation of structured programs such as mindfulness-based stress reduction (MBSR). The value of MBI to tackle some mental health problems was demonstrated (Hoge et al., 2023). MBI using face-to-face groups of medical students were shown to decrease stress, anxiety, and depression and improve academic success (Lampe and Müller-Hilke, 2021). Online programs, namely meditation apps, are helpful for university students due to their high motivation to use digital technologies and some reluctance from personal appointments due to time and place constraints and fear of stigmatization or shame (Clement et al., 2015). Two systematic reviews found evidence that online programs using mindfulness meditations can be useful in reducing perceived stress, increasing empathy and self-compassion, and promoting coping strategies in medical students, but specific and non-esoteric language should be used (Yogeswaran and El Morr, 2021; Ungar et al., 2022). A recent systematic review and meta-analysis showed that mobile mindfulness meditation interventions, using mobile devices such as smartphones and apps, are effective in reducing stress and anxiety, and increases the well-being of university students (Chen et al., 2023). These approaches can be included in the category of “digital mindfulness” (Yosep et al., 2024). A problem of online approaches is the challenge to maintain the motivation of the enrolled participants, since the retention rates continuously decrease from the onset of the programs and the percentage of users who continue to be engaged with an app after 21 days may be lower than 10% (Baumel et al., 2019). Therefore, the effects of motivational strategies sent to the participants considering their initial reasons to access the online resources needs to be studied (Jiwani et al., 2022).

Emotion regulation (ER) is an important factor in emotional well-being (Williams et al., 2018). ER is “the processes by which individuals influence which emotions they have, when they have them and how they experience and express them” (Gross and Muñoz, 1995). An inverse relation between poor ER and some of the abovementioned disorders, namely depression and anxiety, was proposed (Kassel et al., 2007). Effective ER is important to accommodate several socio-emotional stressors (Ma and Fang, 2019). Mindfulness training has a beneficial impact on ER (Robins et al., 2012). Furthermore, high self-reported levels of dispositional mindfulness are related to higher levels of differentiation of one’s emotional experiences reflecting effective ER (Hill and Updegraff, 2012), which may be explained by the increased awareness of mental processes during mindfulness practices (Kemeny et al., 2012).

The student population at the Faculty of Medicine of Porto (FMUP), the second largest Portuguese medical school, was recently found to be having high levels of self- perceived stress (Oura et al., 2020). Although studies, discussed earlier, have explored the benefits of mindfulness interventions and the challenges of online program retention, less is known about how targeted motivational strategies within these programs can specifically influence ER in medical students. We hypothesize that by tailoring motivational messages to students’ initial reasons for seeking mindfulness resources, we can enhance engagement and, in turn, improve their ability to regulate emotions by encouraging consistent practice and deeper engagement with the mindfulness techniques. Given the importance of measures to help medical students manage stress and also considering their skills in the use of online tools, we developed an online app, which consisted of 21 consecutive days of access to short meditations recorded in Portuguese. The app, which provided 21 types of meditation to be practiced by students at our medical school, was named Med@Med and was prepared with the inclusion of professors and medical students to address the specific needs of medical students. This study addresses the gap between improving motivation to use the meditation app Med@Med and the consequent emotional benefits by investigating the effects of targeted motivational strategies within a novel, Portuguese-language online mindfulness intervention (Med@Med) on the ER of medical students. We examined how these strategies impact both engagement with the program and subsequent changes in ER.

## 2 Methods

### 2.1 Ethical considerations

Before the start of the study, a detailed description was submitted to the Ethics Committee of FMUP, and the study was approved (License n° 63/CEFMUP/2022). The students were recruited by sending e-mails to the academic e-mail addresses available in the medical school database. This database contains details of all the students that are currently enrolled in the medical course. The e-mail was sent in October 2022. The e-mail contained a presentation of the Med@Med project and asked student interest to be further contacted. The e-mail contained link to a Google Form with an informed consent, which was collected for each participant. Participants were informed of the anonymity in the treatment of the data and that they were allowed to drop out of the study at any time, with subsequent deletion of all the collected data. The participants were also informed about the conditions to ensure privacy and confidentiality of all the data collected in the study. No financial compensation was provided to the students, as participation in the study did not involve any cost. Participants were also informed that if they were receiving professional psychological support, they should share all the information about the Med@Med project with that health professional and that the research team was available to provide any further information.

### 2.2 Participants

Eligible participants were ≥18-year-old students attending the medical course at FMUP who gave their consent to participate.

**Figure 1:**
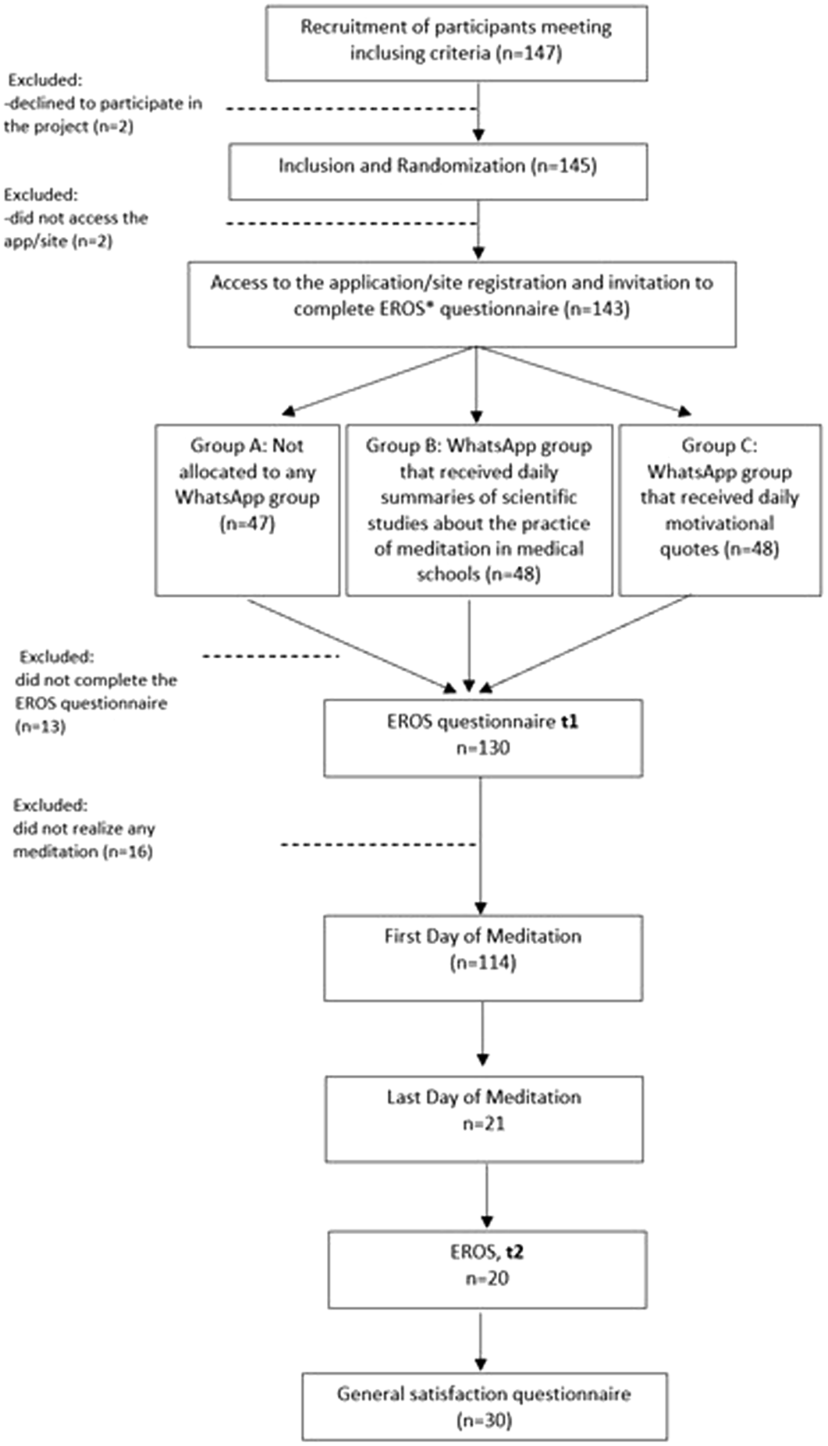
Consolidated Standards of Reporting Trials (CONSORT) diagram of the Med@Med project.

The project Med@Med included two populations of medical students of our medical school. The first group included medical students that replied positively to the e-mail with the invitation and that were willing to use the Med@Med app were included in a longitudinal study that lasted 21 days. Figure 1 presents the Consolidated Standard of Reporting Trials (CONSORT) of the project Med@Med regarding the students that use the Med@Med app. The second group included medical students that did not use the app/site.

For the students that replied in favor to use the app, a questionnaire was sent about the interests and motivations to use a meditation app/site containing 21 distinct meditation exercises during 21 days. The structure of the 21 meditations is shown in Supplementary Table S1. The questionnaire also asked the participants if they had previous experience in the practice of meditation and if they had previously used meditation apps. They were also asked about their motivation to participate in the Med@Med project (stress, anxiety, academic success, sleep, or others) and, on a scale of 1 to 10, what was their expectation to improve that parameter.

We also asked the students if they agreed to be included in a WhatsApp group that receives daily motivational sentences. Herein, according to the answers, the students were distributed in three groups: group A was not included in a WhatsApp group and included the students that explicitly stated that they were not interested in participating in a WhatsApp group, whereas the remaining students were distributed in a random manual manner into two additional groups (groups B and C). Group B received a daily WhatsApp message with a short summary of a scientific study about the advantages of practicing meditation in reducing stress, anxiety, and improving academic performance and group C also received a daily motivational message in a lay language that translated the main results of the scientific study presented to Group B. The messages were sent simultaneously to participants of groups B and C during the first 7 days of the project. The motivational messages Supplementary Table S2. Herein the randomization was determined by the answers of each student who elected the preference about receiving a daily motivational message. The persistence in the use of the app/site of each group was analyzed in the three groups by comparing the number of daily meditations.

### 2.3 Emotional parameters

The emotional parameters used in the longitudinal study of the medical students that used the Med@Med app were accessed by the Emotional Regulation of Others and Self (EROS Questionnaire), selection of an emotion before and after accessing each meditation, and emotional thermometers (ET), as described below.

The general layout of the Med@Med app is shown in Supplementary Figure S1. Before accessing the first meditation, the participants were invited to reply to the EROS questionnaire, available at the app/site. The participants were invited to reply again to the EROS questionnaire after completing the 21 days of the project. The Portuguese version of the EROS (Brandão et al., 2023) questionnaire was used to evaluate interpersonal ER. This self-report scale consists of 19 items and assesses four types of affect regulation: extrinsic affect improving, extrinsic affect worsening, intrinsic affect improving, and intrinsic affect worsening. Items are rated on a Likert-type scale ranging from 1 (not at all) to 5 (a great deal). In the Portuguese study, Cronbach’s α coefficients were 0.88 for extrinsic affect improving, 0.80 for extrinsic affect worsening, 0.83 for intrinsic affect improving, and 0.85 for intrinsic affect worsening.

The students were also asked to select an emotion before (emotion pre-meditation) and after (emotion post-meditation) each meditation. The emotions were identified by subtitled icons (joy, sadness, fear, anger, and disgust) and there was also the possibility of choosing a neutral emotion or not reply to the question (Supplementary Figure S2). The basic emotions were selected based on the work of Ekman (1992). The icons used for each emotion were the characters from the movie *Inside Out* with Portuguese subtitles. At the end of the project, the students had the possibility to express their opinions and satisfaction with the use of the Med@Med app using a Google Form questionnaire, with an open question.

Questionnaires with emotional thermometers (ET) were also used in this study. ET contains five visual analog scales validated for Portuguese (Areia et al., 2020). These scales can be divided into four predictor domains and one outcome domain. The four predictor domains are emotional distress, anxiety, depression, and anger. In these four domains, the user is asked to indicate from 0 to 10 (0: no change; 10: extreme change) the number that describes the degree of emotional change felt during the last week. The result domain corresponds to the need for help. In this last domain, the user is asked to indicate, again from 0 to 10, how much help was necessary to deal with those emotional changes (0: an ability to resolve the previously mentioned changes alone; 10: immediate and desperate need for help). The higher the score in the five domains, the greater the levels of emotional distress, anxiety, depression, and anger and the greater the need for help by the user.

The CONSORT diagram in Figure 1 shows that a total of 147 medical students of FMUP were initially recruited, who were screened for eligibility. Four students (2.7%) were not enrolled, as two failed to access the app/site and two declined to participate. The other 143 participants were randomly assigned to Groups A (*n* = 47), B (*n* = 48), and C (*n* = 48). After excluding 13 participants who did not respond to the EROS questionnaire, a total of 130 participants were included in the study.

The second part of the Med@Med project was directed at students that did not access the app/site. This phase was initiated when the students of the first group were still using the app/site. The students were asked about the reasons for not adhering to the first phase of the project, namely lack of information about the project, lack of time to practice meditation, and aspects such as previous meditation experience and identification with meditative practices, along with an open question. A cross-sectional evaluation was performed to compare the results of the students that accessed the Med@Med app/site with those that did not access because they did not show availability to join the project in the first phase. The two populations were named “Meditation” and “Non-Meditation” and were described as the first and second parts of the project, respectively.

### 2.4 Statistical analysis

The statistical analysis was performed using IBM SPSS Statistics version 28. A paired sample test was used to analyze the difference between the pre-meditation (T1) and the post-21 days (T2) responses in the EROS questionnaire. A chi-square test was used to compare the choices of emotions before and after each meditation session. An independent simple test was used to compare the results of the ET questionnaire between the Non-Meditation group and the Meditation group. The distribution of the continuous data was assessed before choosing the statistical analysis approach. The statistical significance of the tests was assessed at a *p*-value lower than 0.05 (95% confidence interval).

## 3 Results

### 3.1 Participant demographics

Table 1 displays the baseline characteristics of the participants. The majority of participants were females (88.37%), which reflects the gender distribution in our medical school (69.50% females). The mean age (±SD) of the participants was 22.09 years (±3.77). The mean (±SD) year of the MD course was 3.85 (±1.48), which indicates that the project enrolled students from the six-year MD course.

**Table 1:** Demographic characterization of the enrolled medical students enrolled in the Med@Med project, including the students that accessed the meditations and those that did not access.

Baseline features	Students with access to the app/site Med@Med (*n* = 130)	Students that did not access the app/site Med@Med (*n* = 67)
**Gender, *n* (%)**		
Male	*M* = 11.63%	*M* = 20.90%
Female	*F* = 88.37%	*F* = 79.10%
**Other**		
Age (years), Mean (±SD)	22.09 (±3.77)	21.58 (±2.77)
Year at the MD course Mean (±SD)	3.85 (±1.48)	3.51 (±1.64)

### 3.2 Motivations

When the students were asked about the motivations to adhere to the Med@Med project, they were primarily interested in exploring mindfulness meditation as a practice, with 32.7% of participants referring this as their main reason for using the app. Students also reported the need to reduce their stress and anxiety levels (25.2%) or establish a daily meditation routine (15.6%). A small percentage of students (6%) was interested in using meditation to improve their sleep, academic performance (4%), or relationships (3%).

### 3.3 Retention and adherence

By the end of the first 7 days of the project, students in group A (no WhatsApp messages) completed a total of 174 meditations, in group B (scientific messages by WhatsApp) 171 meditations, and in group C (motivational messages in lay language summarizing scientific findings) 208 meditations. Students who received no motivational message (Group A) or only a brief summary of the scientific study (Group B) exhibited similar levels of app usage. A retention rate of 48.3, 56.4, and 67.8% was noted after the first week of the project for the groups A, B, and C, respectively, indicating that the most effective manner to keep the students enrolled was the one used in group C.

**Figure 2:**
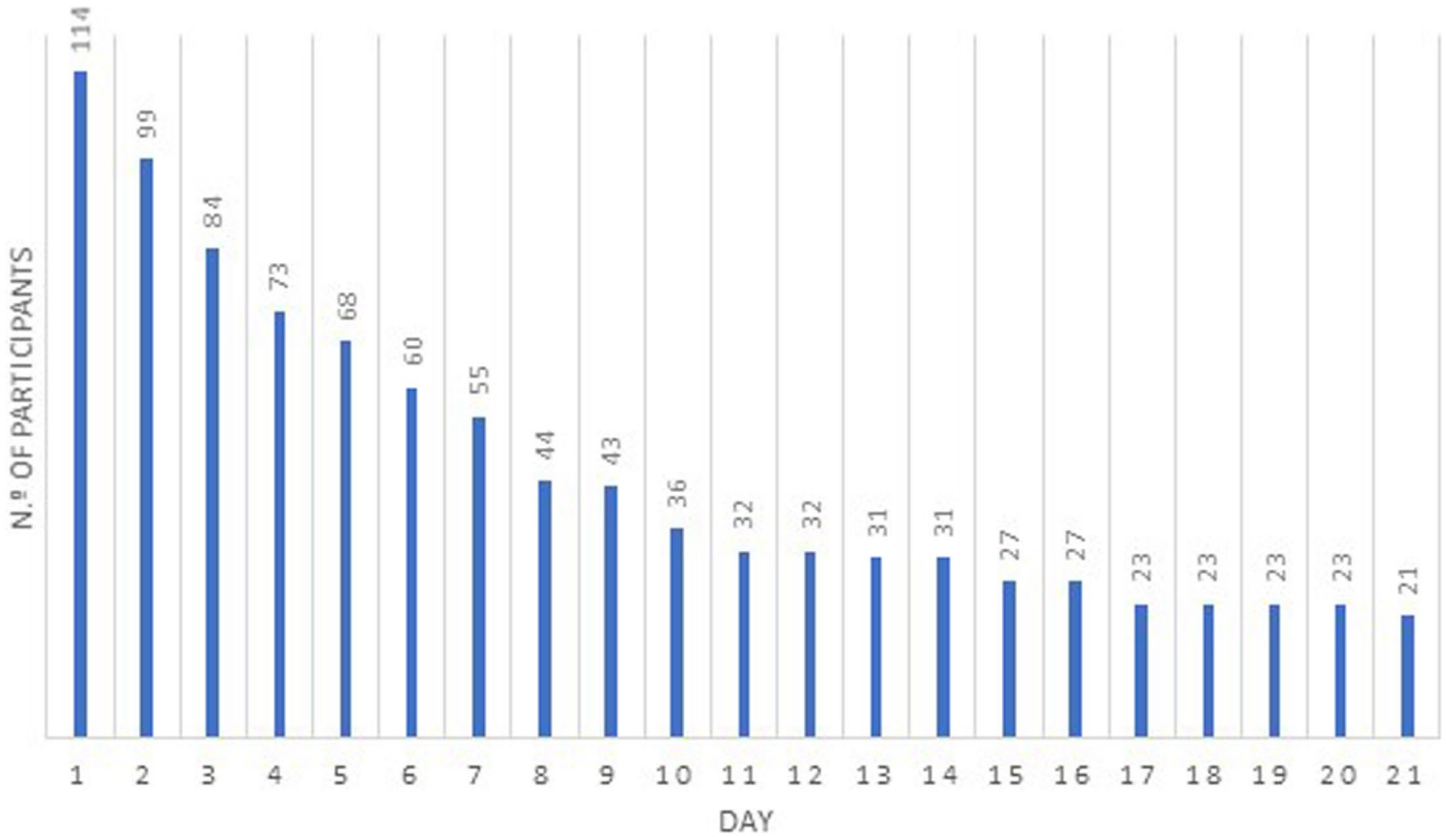
Total numbers of participants in each day of the 21 days of the use of the Med@Med app/site.

At the end of the 21 days, the average number of meditation sessions completed by participants was 8.68. Nearly three-quarters of the participants (73.7%) completed at least three sessions, 31.6% completed more than 10 sessions, with over 100 min of practice. Twenty-one participants completed the 21 sessions, the maximum number of sessions in the program (Figure 2). Twenty participants did not engage in any session.

### 3.4 Emotional parameters

#### 3.4.1 EROS questionnaire

The results obtained from the statistical analysis of the EROS scale at the beginning (T1) and end (T2) of the project are presented in Table 2. There were statistically significant differences in intrinsic affect improving (*t*(19) = −4.20, *p* < 0.001). This implies that the intrinsic affect improving of the participants in the Med@Med project improved from T1 (3.18 ± 0.85) to T2 (3.73 ± 0.67).

**Table 2:** Paired samples statistics of EROS questionnaire, analyzing the differences between T1 (before the first meditation) and T2 (post-21 days).

	Mean	SD	*t*	df	Two-sided p
Extrinsic improving T1	3.80	0.76	−1.08	19	0.294
Extrinsic improving T2	3.92	0.75			
Extrinsic worsening T1	1.38	0.42	1.10	19	0.285
Extrinsic worsening T2	1.28	0.36			
Intrinsic improving T1	3.18	0.85	−4.20	19	<0.001
Intrinsic improving T2	3.73	0.67			
Intrinsic worsening T1	1.96	0.82	1.54	19	0.139
Intrinsic worsening T2	1.78	0.76			

#### 3.4.2 Daily emotions

Table 3 represents the statistical analysis of the variations in the emotions chosen by the participants before and after each meditation session, as shown in Supplementary Figure S2. In the meditation sessions 1 (Introduction: Breathing as an anchor), 2 (breathing and the body), 3 (breathing with rhythms), 4 (deep relaxation breathing), 5 (being with the silence), 7 (body scan – lying down), 10 (The sounds and the silence), and 21 (wrapping session), there were statistically significant differences in the emotions selected by the students. These results indicate that the participants experienced significant emotional changes, namely a decrease in emotions, such as anger and sadness, and an increase in emotions, such as joy. Supplementary Figure S3 shows the variations in each of the 21 days.

**Table 3:** Statistical analysis of the changes in the emotions (pre- vs. post-meditation) in each of the days of the use of the Med@Med app/site.

Emotions	*N*	*p*-value
Day 1	114	0.000086*
Day 2	99	0.000060*
Day 3	84	0.019376*
Day 4	73	0.010082*
Day 5	68	0.006482*
Day 6	60	0.212134
Day 7	55	0.001479*
Day 8	44	0.329970
Day 9	43	0.121622
Day 10	36	0.014972
Day 11	32	0.171258
Day 12	32	0.209143
Day 13	31	0.350140
Day 14	31	0.411735
Day 15	27	0.478991
Day 16	27	0.216292
Day 17	23	0.169541
Day 18	23	0.682421
Day 19	23	0.370612
Day 20	23	0.512168
Day 21	21	0.021932*

*N* represents the number of responses per day. Statistically significant changes (*p* < 0.05) are marked with *.

### 3.5 Emotional thermometers

Seventy students that used the Med@Med app (meditation group) and 62 students that did not access any of the recorded meditation (non-meditation group) replied to the ET questionnaire. The results in Table 4 indicate differences between the groups, with the meditation group showing less emotional distress and anxiety than the non-meditation group. Statistically significant differences in the equality of variances were detected for all emotional thermometers (*p* < 0.001 for emotional distress and anxiety, *p* = 0.12 for depression, and *p* < 0.001 for anger) since students from the meditation group presented significantly lower scores than the non-meditators. Significant differences in the equality of means between the groups for all emotional thermometers were detected except for the depression thermometer, in which the difference was not significant. The 95% confidence interval for the difference in means between the groups was −1.53 to −0.33 for emotional distress, −2.54 to −1.17 for anxiety, −1.11 to 0.29 for depression, and −0.51 to 0.91 for anger.

**Table 4:** Statistical analysis of the emotional thermometers (ET) questionnaire between the non-meditation (*N* = 62) and the meditation (*N* = 70) groups.

	Group	Mean	SD	*t*	df	Two-sided p
ET emotional distress	Mediation	3.46	1.03	−2.97	82.86	0.004
	Non-meditation	4.39	2.27			
ET anxiety	Mediation	3.87	1.14	−5.12	80.85	<0.001
	Non-meditation	5.73	2.64			
ET depression	Mediation	3.43	1.17	−1.11	130	0.269
	Non-meditation	3.84	2.68			
ET anger	Mediation	4.11	1.12	0.52	129	0.604
	Non-meditation	3.92	2.75			

### 3.6 Satisfaction with the app

The post-intervention survey to assess the satisfaction of the medical students with the use of the Med@Med app/site was completed by 30 participants. The majority of the students (60%) used the app version of the project.

When asked about the reasons for not completing the full project, 43.8% of the participants said that they had forgotten to continue, while 12.5% referred to the lack of persistence and 12.5% reported lack of time. Almost all the participants (96.7%) expressed the intention to continue practicing meditation in the future, indicating a strong interest in incorporating mindfulness practices into their daily routine. When asked if they would recommend the Med@Med project to a friend, 93.3% of the respondents answered affirmatively, indicating a high level of satisfaction with the program. In an open question, the students were invited to give their opinion about the project. Nineteen participants provided feedback that emphasized the physical and emotional benefits they experienced through a regular meditation practice. Some reported greater ease in identifying and accepting their emotions, while others noted improvements in emotional management and overall well-being. Several participants mentioned that the project helped them establish a meditation routine and chart their personal evolution over time. One student expressed disappointment with the program, but no specific reasons were provided.

### 3.7 Non-participants in the meditation part of the Med@Med project

A Google Form was sent to those medical students that did not participate in the Med@Med project. We expected to obtain information about the reasons that influenced their decision. As referred to above, the Google form also included the EROS questionnaire and emotional thermometers—three scales that were evaluated through the app/site, which helped us to compare the “meditators” with the “non-meditators” groups.

The survey was answered by 67 students, with the majority (79.1%) being females and the average age being 21.58 years. Among these respondents, 68.7% had no previous experience with meditation practice.

The results revealed that the most common reason for not participating in the project was a lack of knowledge of the project, with 61.9% stating that they did not know about the project. Forgetfulness was another factor, with 15.9% reporting that they forgot to participate. Lack of time was cited by 11.1% of the respondents, while 7.9% said that they did not identify with the practice of meditation. A small percentage (1.6%) mentioned that they were afraid of the consequences of meditation.

## 4 Discussion

The current Med@Med project explored the feasibility of the development and use of a meditation app prepared in Portuguese for medical students. The enrollment of a student who was in the last year of the MD course was important for gaining a broader perspective of the challenges that students face during the course. The project Med@Med had two main goals. The first was to analyze the motivations of medical students to use a mindfulness meditation app and evaluate their persistence throughout the duration of the project. The second aim was to establish emotional benefits of the use of the app, which could help the students to deal with the challenges of a medical course (Dyrbye et al., 2006; Tam et al., 2019). This project was also primarily performed considering the high self-perceived stress levels of our medical students (Oura et al., 2020) and the success of an elective and practical course about mindfulness and contemplative neurosciences, which has been functioning since 2020 with short groups (12 per course) of FMUP medical students. It could be relevant to introduce meditation and mindfulness practices to medical students who could not participate in this study.

Regarding the first aim, the main motivations of the students to participate in the project were to “experience a mediation app for the first time,” “decrease stress/anxiety,” or “implement a routine of meditation.” The overall motivation to adhere was high. The survey that evaluated why medical students did not adhere to the Med@Med project showed that the reasons were not related with meditation since only a few students replied that they did not identify with meditation practices or were afraid of the consequences of meditation. In fact, most of the students did not adhere to the project because they were not reached by the methods used to contact the students (e-mail or Facebook posts). Using additional resources like Instagram posts, distribution of flyers, and posting posters in areas attended by the students or organizing small meetings to present the Med@Med project will be used in future editions of the project to increase the participation of the students. Curiously, there was a gender difference between the groups that was enrolled in the project (“meditators”) and the group that did not participate (“non-meditators”), the latter gathering more male students. This gender bias may be explained by previous studies showing that female medical students are more prone to report self-stress and seek coping strategies (Madhyastha and Kamath, 2014) whereas males are usually harder to recruit for health behavior research (Ryan et al., 2019). A high female participation among the medical students recruited for the projects was expected since the large majority (about 70%) of our medical students are females. Collectively, the results indicate that the FMUP medical students are interested in the use of mindfulness meditation apps, but the project advertising should be considerably improved.

Based on the main motivations of the students that intended to participate in the Med@Med project, a course including 21 daily short-guided meditations was developed. The duration of the program (21 days) was determined considering some recent studies about the time of persistence in meditation programs (Lam et al., 2023), namely studies showing that the proportions of users that download apps and actually used them dramatically decreases after 21 days (Baumel et al., 2019). We also evaluated the effect of motivational WhatsApp messages prepared taking into account relevant studies about the benefits of meditation for medical students. The group that received a “translation” of the scientific results in a lay language (group C) was more inclined to continue to use the app whereas the group that received a short scientific message had a decrease in use similar to the group that did not receive any WhatsApp message. In spite of the low numbers of students, this is an interesting result since one could expect that the students of a scientific course, such as an MD, would be receptive to scientific communication. We should also recognize that medical students are frequently overloaded with facts/information and the motivational messages using a scientific explanation could have been misunderstood as additional information. Nevertheless, it should be noted that the retention rates along the 3 weeks of the Med@Med project were higher than other meditation apps (Baumel et al., 2019). When the students that were enrolled in the project were anonymously asked about the reasons to not complete the 21 days of meditation, 69% reported forgottenness, lack of time, or considered themselves as poorly persistent. In the open question, it was revealed that as only one meditation session was available per day, it was a reason to discontinue the practice. As mentioned earlier, the release of one meditation per day was an option to increase the possibility of building a meditation routine but several students reported that they would prefer to freely navigate through the app and perform more than one session daily. This aspect needs to be considered in future editions of the Med@Med project. The students that used the Med@Med app referred that they were likely to participate in future editions of the Med@Med project and to recommend their colleagues/friends to also participate, which indicates a satisfaction with the Med@Med app. The motivational messages to use the meditation app could be improved, but this would not stimulate intrinsic motivation. Factors related to the reason action approach, which aims to establish the reasons why the subjects adhere to health behaviors, along with aspects of the theory of planned behavior, which proposes that intentions drive behavior, should be used in more detail in the initial characterization of the medical student population enrolled in a future edition of the Med@Med project (Crandall et al., 2019). This approach has recently provided interesting results regarding the predictions to stick with meditation practices (Crandall et al., 2019), that is, analyzing the reasons to persist in the use of a meditation phone app by students, showed that no prior experience with meditation along with expectations regarding the practice were the best predictors of the persistence to use a mediation app. Changing the app to allow a sequential but free navigation and improving the characterization of enrolled students after the initial recruitment could increase the retention rates of the project Med@Med. Additionally, and as referred below, disclosing the emotional benefits of the use of the Med@Med app/site that were established by the current exploratory study may also be important to motivate medical students to participate and persist in the use of the Med@Med app in future editions. A final aspect to consider is the time of the day to motivate our students to use the app. Since practicing meditation in the morning was associated with higher rates of maintaining a meditation practice with the app (Berardi et al., 2023), we need to send the motivational messages in the morning.

The second aim of the project was to evaluate the effects of participating in the 21 meditation sessions in parameters related to emotions. The comparison of the students before and after completing the sessions, in the case of the EROS questionnaire, supported the effectiveness of the use of the Med@Med app in enhancing ER, especially improvement in the intrinsic affect. Intrinsic affect, which refers to the degree of positive or negative feelings experienced by an individual, is a critical component of ER. The improvement of intrinsic affect indicates that the participants were able to regulate their emotions more effectively and suggests that they were able to experience more positive emotions at the end of the Med@Med project. The analysis of the identification with basic emotions before and after each practice by selecting an icon or not identifying with any icon, in a manner similar to widely used meditation apps like Insight Timer, also provided interesting results, since the general trend was to a shift from negative (anger, sadness) to positive emotions (joy) or not identifying with any emotion (neutral emotion). The change in the distribution of identification with emotions was statistically significant in the initial and final sessions, but also in the focus session related to breathing practices, body scan, or sensorial practices (sounds and silence). Although we cannot demonstrate that the emotional improvement was due to each specific practice per se, these results reinforce the results obtained with the EROS scale. Collectively, the results of the use of the Med@Med app indicate the potential benefits of incorporating mindfulness meditation into daily practices in our medical school.

With regard to the other parameters analyzed, namely emotional thermometers, we compared the medical students that meditated with those that did not meditate. There were statistically significant differences between the groups regarding the emotional thermometers with the meditation group significantly reporting less emotional distress and anxiety than the non-meditation group, whereas no differences were detected in the depression thermometer. The lack of longitudinal evaluation of the results of the emotional thermometer demands caution in the analysis, but the results are interesting inasmuch as the prevalence of females in the meditator group, as explained earlier. In fact, female medical students tend to report higher emotional distress (Madhyastha and Kamath, 2014), whereas our meditators group reported the opposite. Furthermore, women benefit more than men from meditation training (Rojiani et al., 2017; Upchurch and Johnson, 2019), which may also have accounted for the differences in meditation and non-mediation population. These aspects need to be evaluated in a future edition of the project.

## 5 Strengths and limitations of the study

The present study is innovative and seeks to understand the needs and motivations of the students of the second largest Portuguese medical school. The recording of the meditations in Portuguese and specifically conducted with language appropriated to medical students can also be considered a strength of the present study. Although the results cannot be extrapolated to other medical schools, Med@Med can be a useful tool for the large Portuguese-speaking community throughout the world. It should, however, be noted that the gender bias in Portuguese medical schools, as the students are largely female, may determine constraints in adherence and effects in medical schools where the students are mostly males. Future studies using a larger sample of students at our medical school and also expanding to other medical schools that use Portuguese as their primary language may be relevant.

One decision when developing the app was to deliver one mediation per day, instead of allowing a free use of the meditation app. This decision probably accounted for a decrease in the retention rate, as explained by some students. In the next edition, we will deliver the 21 meditations when the Med@Med is available and we will evaluate if the rate of retention increases. One limitation regarding research in the use of mediation apps is the selection of questionnaires to evaluate the effects of the practice and whether replying to questionnaires should be mandatory to be enrolled in the study, as we decided with the EROS questionnaire as a condition to first access the app/site Med@Med.

Our medical school population frequently complains about being excessively recruited to reply to multiple and long questionnaires. The use of a simple icon to allow a self-identification with an emotion was used in the Med@Med app to allow to get some feedback about the effects of each practice, in a manner similar to what is done in registered meditation apps such as Insight Timer, but the scientific value of such measures was not studied in detail, to our best knowledge. Furthermore, we cannot discard the fact that a self-report of emotions based on the use of icons may have different meanings to different persons. Mixed-methods analysis may be useful to evaluate the effects of Med@Med app and also simple and more objective tests to evaluate stress like measuring cortisol from saliva could be considered (Hearn and Stocker, 2022). Besides the importance of objective measures of stress such as cortisol, it is may be important to evaluate other objective parameters such as the self-reported quality of life and also changes in the academic achievements of the students. Another aspect that could be a confounder of the effects is prior exposure to meditation practices and whether the students have regular access to other meditation apps. This could have influenced both the adherence and engagement and also the emotional effects of the app Med@Med. Furthermore, there are important aspects of well-being that were not controlled, such as physical exercise and spiritual practices, which may also have played a significant role. Future studies should be designed as a randomized controlled trial using active controls. Finally, since this is the first study, to our knowledge, that developed a mindfulness mediation app in Portuguese for medical students and the results are promising in terms of emotional benefits, the present study may be particularly relevant to fostering promotion of health in the medical students, which may have impact in their future life as medical doctors. The long-term implication of this study will be determined in our medical school with a specific focus of the students of the last year of the medical course who face emotional challenges in the preparation for the exams to access the medical internship.

## 6 Conclusion

The project Med@Med demonstrates the importance of understanding the motivations behind medical students’ engagement with meditation apps by creating a meditation app that meets the needs of the participants. The new meditation program helped our medical students to improve their well-being and foster emotional regulation. The project will be continued by establishing more effective measures to recruit more medical students and reinforce motivational strategies to persist in the use of the app in order to improve emotional regulation and decrease stress and anxiety. Due to its positive impact on mental health and sleep of physician assistants when using app-delivered mindfulness meditation (Smith et al., 2021), the full development of a free version of the Med@Med app may be also available after graduation for our medical students. An important aspect of the Med@Med app is that it is totally prepared in Portuguese language. Apart from medical students of our faculty, we have several students from countries that have Portuguese as their official language, mainly from Guiné Bissau, Mozambique, and East Timor, and these students have frequent challenges of underachievement, in a manner similar to what occurs in other European countries (Singh Grewal et al., 2024), along with other problems that pose additional challenges. Many of those students have clear difficulties in managing English, the language used in most of the apps of mindfulness meditation interventions. Another population that could benefit from our Med@Med app is outside our medical school. Many other Portuguese-speaking medical schools have also identified stress as a major concern for the students, for example in Brazil (Damiano et al., 2021). Therefore, there are multiple possibilities to use a meditation app recorded in Portuguese outside our medical school.

## Data Availability

The datasets presented in this study can be found in online repositories. The names of the repository/repositories and accession number(s) can be found in the article/Supplementary material.
