# Supplementary material for: Developing and evaluating a Portuguese-language meditation App for medical students: motivation, adherence, and emotional effects
Source: Front Psychol. 2025 Mar 5;16:1422205. doi: 10.3389/fpsyg.2025.1422205 (PMC11922080; doi:10.3389/fpsyg.2025.1422205)
Supplement: Supplementary file 1 [file Data_Sheet_1.pdf]

Supplementary Material

Developing and evaluating a Portuguese-language meditation App for medical students: motivation, adherence, and emotional effects

Ana Rita Soares[1], Sandra Soares[2], Tânia Brandão[3], Ricardo João Teixeira[4,5], Isaura Tavares[1,6*]

1- Department of Biomedicine, Unit of Experimental Biology, Faculty of Medicine, University of Porto, Portugal

2 - Dr. Manuel Gomes de Almeida School Cluster – AEMGA, Vila Nova de Gaia, Portugal

3 - William James Center for Research, ISPA – Instituto Universitário, Lisbon, Portugal

4 - University of Coimbra, Faculty of Psychology and Educational Sciences, CINEICC, Coimbra, Portugal

5- REACH - Mental Health Clinic, Porto, Portugal

6 - I3S-Instituto de Investigação e Inovação em Saúde, Universidade do Porto, Portugal

*** Correspondence:**
Isaura Tavares
isatav@med.up.pt

1 Supplementary Tables

Supplementary Table 1: Structure of the 21 days meditation course, with titles of each meditation session.

Day	Session title
1	Introduction. Breathing as an anchor
2	Breathing and the body
3	Breathing with rhythms
4	Deep Relaxation Breathing
5	Being with the silence
6	Body conscious-sitting
7	Body scan- lying down
8	Body- Conscious stretching
9	Body- Conscious eating
10	The sounds and the silence
11	Grounding
12	Gratitude
13	Letting go
14	Identifying emotions
15	Naming emotions in a non-reactive manner
16	Equanimity in emotions

17	Compassion
18	Self-compassion
19	Identifying thoughts
20	Observing the structure of thoughts
21	Wrapping session

Supplementary Table 2: Daily messages used in the 2 Whatsapp groups. Group B received a summary of scientific studies in lay language whereas group C received a motivational message based on the main findings of the scientific study used in that day for group B.

Group B – Scientific communication

Days	Quotes
**-1**	Ready to use the Med@Med app?
**0**	Tomorrow starts the first of 21 days of meditating together. Get ready!
**1**	Scientific studies show that we can increase our concentration through the practice of various types of meditation. (https://doi.org/10.1080/13674679908406332)
**2**	Systematic reviews show improvements in self-perceived stress by medical students involved in online mindfulness courses. (https://www.tandfonline.com/doi/full/10.1080/10872981.2022.2082909)
**3**	Partcipation in virtual programs that use meditation improves medical students ‘exam scores (https://slejournal.springeropen.com/articles/10.1186/s40561-021-00166-7)
**4**	There is scientific evidence that online mindfulness interventions are beneficial for medical students´mental health. (https://bmcpublichealth.biomedcentral.com/articles/10.1186/s12889-021-12341-z)
**5**	Scientific studies show that medical students, with few free time, benefit from participating in short meditation courses. (https://link.springer.com/article/10.1007/s40670-019-00708-2)
**6**	There is evidence that the physical and social isolation of medical students is a predictive factor for the development of mental illness. (https://www.mdpi.com/1660-4601/19/18/11496)
**7**	Population studies of first-year medical students show levels of stress far greater than those of other college students. (https://journals.plos.org/plosone/article?id=10.1371/journal.pone.0240667)

Group C – Motivational communication

Days	Quotes
**-1**	Ready to use the Med@Med app?
**0**	Tomorrow starts the first of 21 days of meditating together. Get ready!
**1**	Do you want to be more focused on college? Practice meditation!

**2**	You will feel less stressed if you practice meditation.
**3**	Did you know that practicing meditation improves exam results in medical students?
**4**	You may improve your mental health if you practice mindfulness meditation.
**5**	You just need to practice a few minutes of meditation to benefit from stress management in college! Don´t give up
**6**	By joining the Med@Med project, you are becoming part of a community. It is a new way to promote connections within FMUP. Congratulations on being with us.
**7**	Getting to Medical School can be stressful. But this is not a fatality: take care of yourself!

Supplementary Figures

Supplementary Figure 1- Screenshots of the app/site Med@Med showing initial images of the App/Site Med@Med. The app is totally written in Portuguese and has introductory sentences. "The art of meditation. Meditation practices require intention and practicing meditation requires some persistence. Before and after each meditation you will find icons with 5 basic emotions. Please elect the icon representing the emotion that better describes/identifies the present moment".

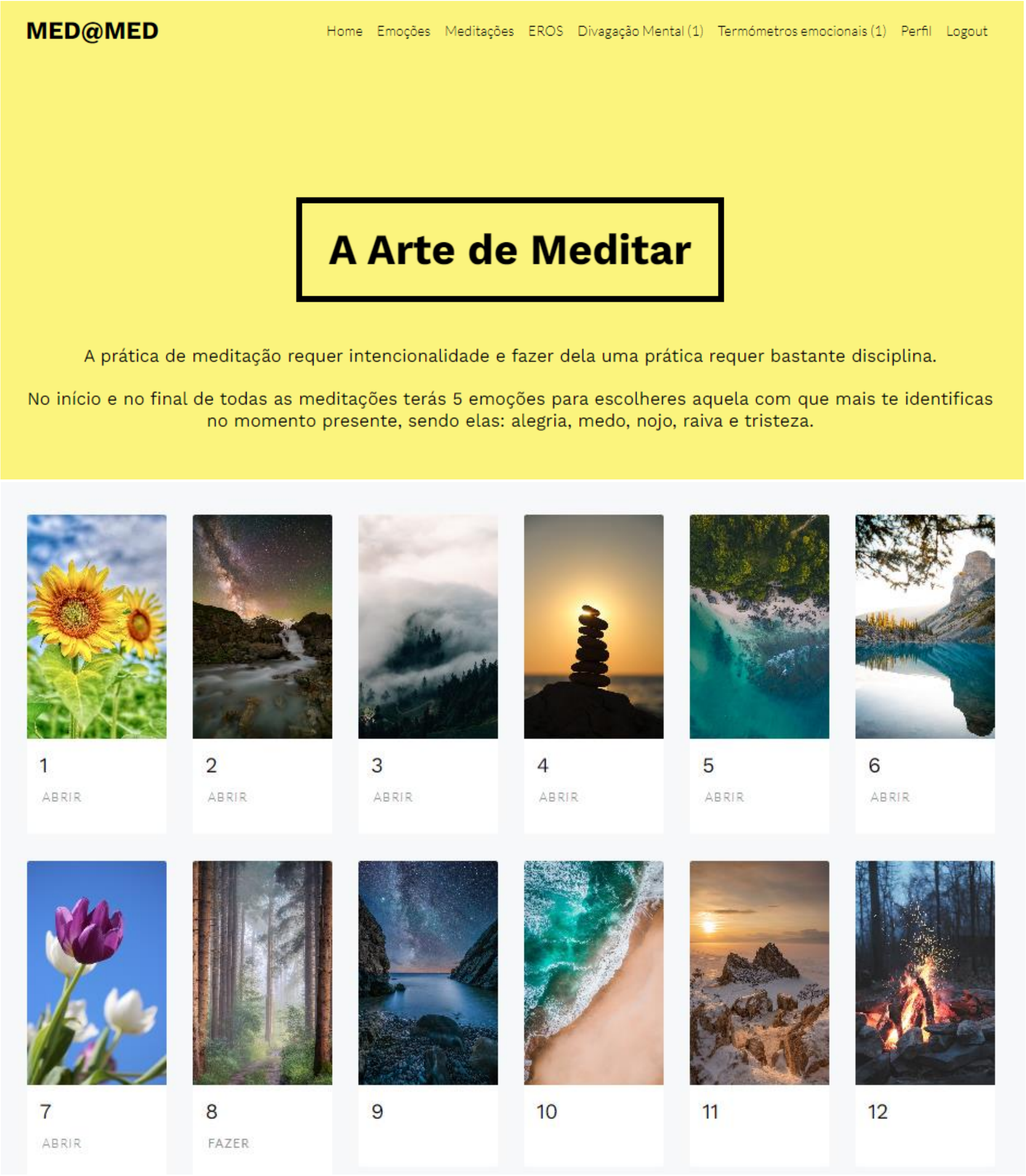
MED@MED
Home Emoções Meditações EROS Divagação Mental (1) Termómetros emocionais (1) Perfil Logout
A Arte de Meditar
A prática de meditação requer intencionalidade e fazer dela uma prática requer bastante disciplina.
No início e no final de todas as meditações terás 5 emoções para escolheres aquela com que mais te identificas no momento presente, sendo elas: alegria, medo, nojo, raiva e tristeza.
1 ABRIR
2 ABRIR
3 ABRIR
4 ABRIR
5 ABRIR
6 ABRIR
7 ABRIR
8 FAZER
9
10
11
12

Supplementary Figure 2- Changes in election of icons of emotions before and after each meditation. In **A** icons used to identify the basic emotions, based in the movie "Inside Out", namely Joy (Alegria), Sadness (Tristeza), Fear (Medo), Anger (Raiva) and Disgust (Nojo), namely at day 8 (Conscious Stretches). The possibility of "No Answer" or Neutral emotion was also considered.

In **B** an example of a student that identified Anger as the initial emotion ("Emoção inicial") and passed to Joy after the meditation ("Emoção final") at day 8 (Conscious Stretching).

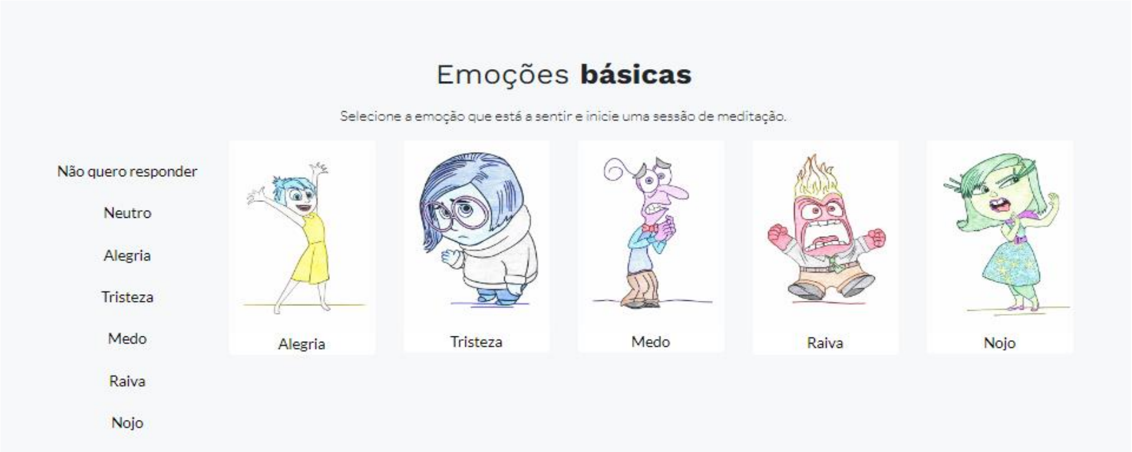
Emoções **básicas**
Selecione a emoção que está a sentir e inicie uma sessão de meditação.
Não quero responder
Neutro
Alegria
Tristeza
Medo
Raiva
Nojo
Alegria
Tristeza
Medo
Raiva
Nojo

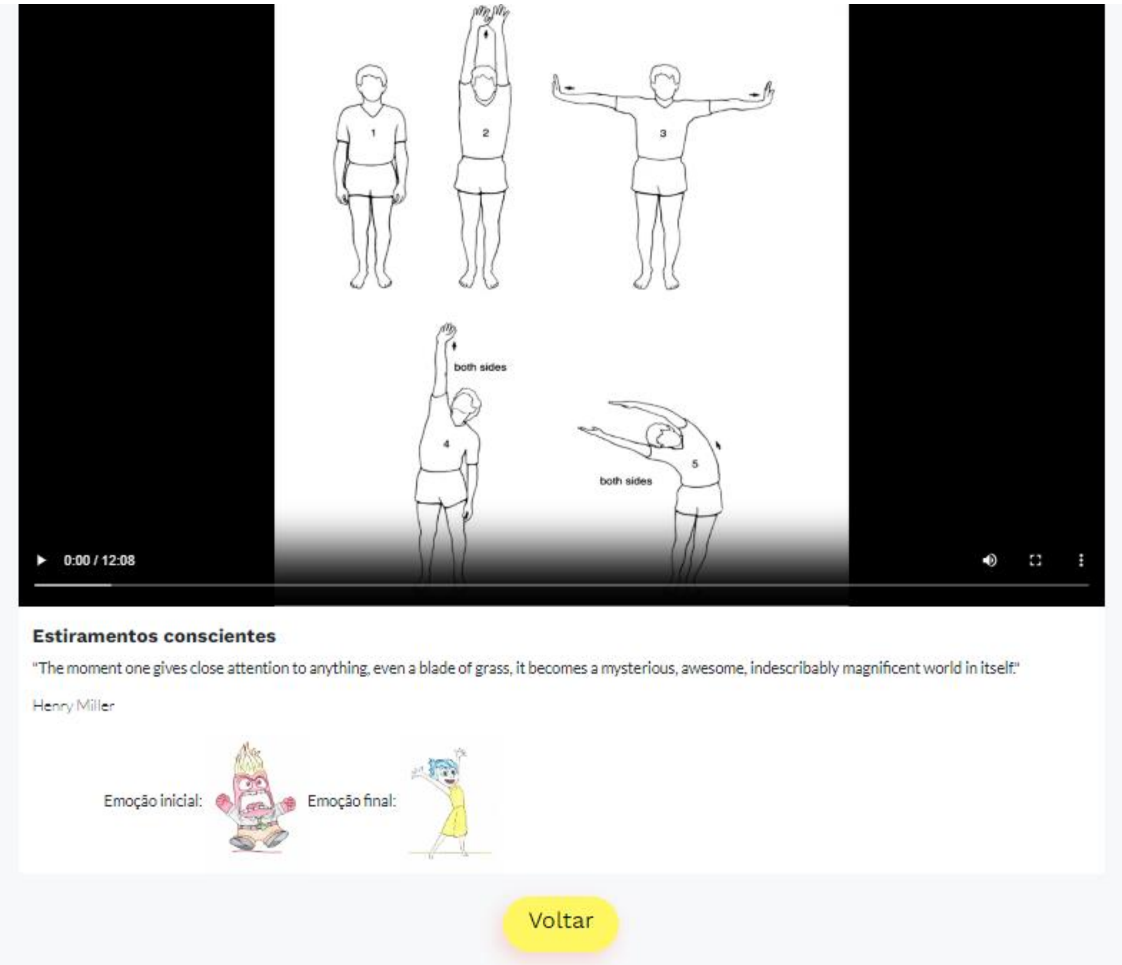
1
2
3
both sides
4
both sides
5
0:00 / 12:08
Estiramentos conscientes
"The moment one gives close attention to anything, even a blade of grass, it becomes a mysterious, awesome, indescribably magnificent world in itself."
Henry Miller
Emoção inicial:
Emoção final:
Voltar

Supplementary Figure 3- Changes in election of icons of emotions before (Pre-mediation) and after (Post-meditation) in the days when statistically significant differences were obtained. Data from Table 3, including p values.

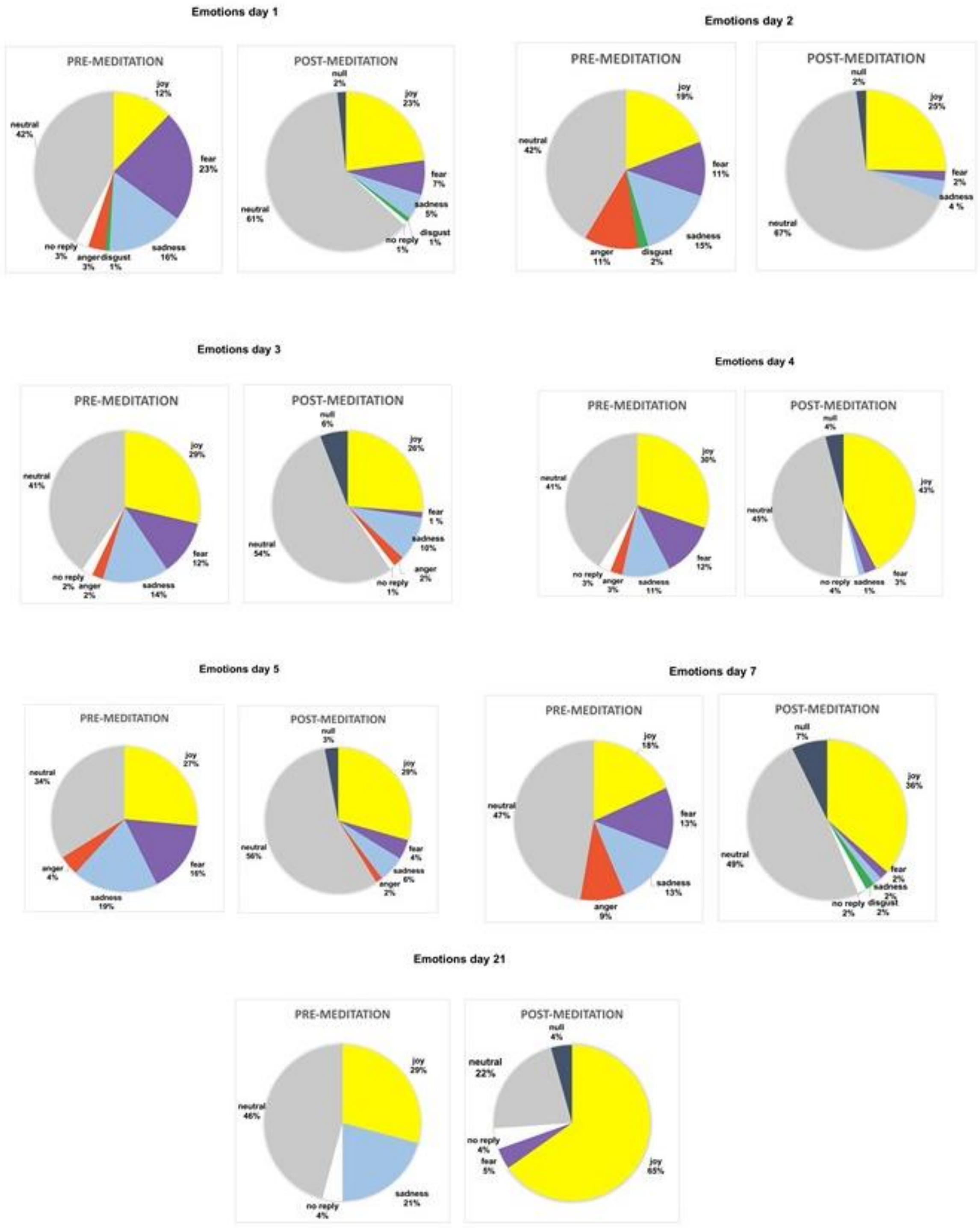
Emotions day 1
PRE-MEDITATION
joy 12%
fear 23%
sadness 16%
disgust 1%
anger 3%
no reply 3%
neutral 42%
POST-MEDITATION
null 2%
joy 23%
fear 7%
sadness 5%
disgust 1%
no reply 1%
neutral 61%
Emotions day 2
PRE-MEDITATION
joy 19%
fear 11%
sadness 15%
disgust 2%
anger 11%
neutral 42%
POST-MEDITATION
null 2%
joy 25%
fear 2%
sadness 4 %
neutral 67%
Emotions day 3
PRE-MEDITATION
joy 29%
fear 12%
sadness 14%
anger 2%
no reply 2%
neutral 41%
POST-MEDITATION
null 6%
joy 26%
fear 1 %
sadness 10%
anger 2%
no reply 1%
neutral 54%
Emotions day 4
PRE-MEDITATION
joy 30%
fear 12%
sadness 11%
anger 3%
no reply 3%
neutral 41%
POST-MEDITATION
null 4%
joy 43%
fear 3%
sadness 1%
no reply 4%
neutral 45%
Emotions day 5
PRE-MEDITATION
joy 27%
fear 16%
sadness 19%
anger 4%
neutral 34%
POST-MEDITATION
null 3%
joy 29%
fear 4%
sadness 6%
anger 2%
neutral 56%
Emotions day 7
PRE-MEDITATION
joy 18%
fear 13%
sadness 13%
anger 9%
neutral 47%
POST-MEDITATION
null 7%
joy 36%
fear 2%
sadness 2%
disgust 2%
no reply 2%
neutral 49%
Emotions day 21
PRE-MEDITATION
joy 29%
sadness 21%
no reply 4%
neutral 46%
POST-MEDITATION
null 4%
neutral 22%
no reply 4%
fear 5%
joy 65%
